# Adolescents’ perceptions of standardised cigarette packaging design and brand variant name post-implementation: a focus group study in Scotland

**DOI:** 10.1186/s12889-019-7552-0

**Published:** 2019-09-05

**Authors:** Danielle Mitchell, Crawford Moodie, Nathan Critchlow, Linda Bauld

**Affiliations:** 1 Institute for Social Marketing, Faculty of Health Sciences and Sport, University of Stirling, Stirling, FK9 4LA Scotland; 2 Usher Institute, College of Medicine and Veterinary Medicine, University of Edinburgh, Edinburgh, EH8 9AG Scotland

**Keywords:** Standardised packaging, Adolescent smoking, Focus groups

## Abstract

**Background:**

The United Kingdom (UK) fully-implemented standardised packaging for cigarettes and rolling tobacco on 20th May 2017. We explore adolescent’s awareness of, and responses to, standardised cigarette packaging in the UK after it became mandatory.

**Methods:**

Eight focus groups were conducted in schools in Scotland with 16–17 year-olds (*n =* 41), between November 2017 and November 2018, to explore awareness of, and responses to, standardised cigarette packaging. Unlike in Australia, where only straight-edged flip-top cigarette packs are permitted, in the UK standardised cigarette packs can have slim designs, and different edge types (straight, rounded or bevelled) and opening styles (flip-top or shoulder box). We explored how each of these pack formats was perceived. We also explored to what extent brand variant name differentiated cigarettes sold in standardised packaging.

**Results:**

Most participants were aware of standardised packaging without being shown pack stimuli. Standardised packs were considered embarrassing and off-putting, and the health warnings salient. Among the standardised packs shown, there was a preference for the slimmer pack, viewed as more discrete and the cigarettes potentially less harmful, and the shoulder box, considered cool and different. Participants were interested in some brand variant names on standardised packs (e.g. Legendary Black), particularly those they considered to imply coolness and sophistication.

**Conclusion:**

Adolescents consider standardised cigarette packs in the UK unappealing, and the warnings salient, two core aims of this measure. However, positive reactions to some of the standardised packs (slimmer pack, shoulder box), and variant names used, has implications for countries developing standardised packaging regulations.

## Background

Tobacco packaging is a marketing tool that can influence smoking-related attitudes and behaviours [1–5]. In response, the United Kingdom (UK) became the third country, following Australia (2012) and France (2017), to fully-implement standardised (or plain) packaging for cigarettes and rolling tobacco [6]. Three more countries have since introduced standardised packaging (New Zealand, Norway, Republic of Ireland), and it is being considered in several other countries [7]. In the UK, standardised packaging was implemented alongside the Tobacco and Related Products Regulations 2016 [8], and both became mandatory 20th May 2017, after a 12-month transition period. The legislation stipulates that all cigarettes and rolling tobacco must be sold in drab brown packaging, contain a minimum of 20 cigarettes or 30 g for rolling tobacco, have pictorial health warnings covering at least 65% of the primary surfaces and text warnings covering at least 50% of the secondary surfaces, and have no promotional features (e.g. logos, inserts, price-marking or promotional offers) or features which indicate reduced harm, lifestyle or environmental benefits, or reference taste, smell, flavours or additives [6, 9].

Although six countries have fully-implemented standardised packaging, there are key legislative differences. Concerning pack structure, in Australia and New Zealand all cigarette packs must be straight-edged with a flip-top lid, with slimmer packs banned, whereas in the UK (and France, Norway and Ireland) tobacco companies are permitted to sell bevelled or rounded-edged packs, shoulder boxes, and slimmer packs [10]. This may have implications for consumer behaviour, as internal tobacco industry research and marketing documents claim that packaging which diverges from the ‘traditional’ style (e.g. bevelled-edges and unique opening methods) is viewed positively and considered ‘elegant’ or ‘classy’ [11]. Similarly, academic research with young adults exploring perceptions of standardised packs which differ by edge type, opening style or pack size has found certain pack features, such as bevelled-edges, novel opening styles and slimmer formats, to increase appeal [12–14]. No study, however, has explored consumers’ perceptions of, and attitudes to, these types of pack features in a market where standardised packaging has been introduced.

Aside from changes in pack structure, brand variant name provides another opportunity for tobacco companies to differentiate products sold in standardised packaging [15]. Market research suggests that brand name can influence product perceptions [16, 17]. For cigarettes, research suggests that brand name has the ability to communicate desirable traits such as popularity (e.g. cigarette brand Kool), particularly among young people [18]. In Australia, three-quarters of brand variant names contained a colour descriptor after standardised packaging was implemented, compared to less than a half before it was required [19]. As colour is one of the most influential aspects of packaging [20], the amplified use of colour descriptors once standardised packaging had been introduced suggests that tobacco companies may be using brand variant names to communicate product features or invoke memories of the fully-branded packaging. Only one study has explored how consumers respond to brand names on cigarette packaging in a market where standardised packaging has been introduced. Using a taste test, smokers were randomly allocated to smoke a cigarette from one of two standardised cigarette packs, one of which had a premium brand name and one a value brand name - the cigarettes contained in both packs were identical [21]. Smokers considered cigarettes from the pack with a premium brand name (e.g. Peter Stuyvesant) to be better tasting and less harsh than from the pack with the value brand name (e.g. JPS Blue).

Only one study in the UK has explored adolescent’s awareness of standardised packaging, 10 months into the transition period (March 2017). The online survey found that only one in five adolescents were aware of standardised packaging [22]. No qualitative study in the UK, or indeed in any country that has introduced standardised packaging, has explored adolescent’s awareness of, and response to, this policy after it has been fully-implemented. It is important to do so given that smoking initiation is often before the age of 18 [23], and standardised packaging is predicted to have a particular impact on youth [24]. We explored to what extent (if at all) adolescents were aware of standardised packaging, and how it was perceived. We also explored their reactions to the different pack structures permitted for standardised packs in the UK (e.g. slim packs and shoulder boxes), and the role of brand variant names as a mechanism to differentiate products or create appeal.

## Methods

### Design and participants

Eight focus groups were conducted in schools in Scotland with 16–17 year-olds (*n =* 41), between November 2017 and November 2018, to explore awareness of, and responses to, standardised cigarette packaging. Groups were segmented by gender and smoking status (never-smoker, ever-smoker), determined by a brief questionnaire given to students in class, to allow us to explore any differences between these groups. This questionnaire was based on items used in previous studies of tobacco control with adolescents in the UK [14]. Participants were provided with the following options: ‘I have never smoked, not even a puff or two’, ‘I have smoked a few times before’, ‘I smoke at least once a month’, ‘I smoke at least once a week’ and ‘I smoke every day’. Those who selected ‘I have never smoked not even a puff or two’ were categorised as never-smokers, and those who selected any other option were categorised as ‘ever-smokers’ [14, 25]. Three ever-smoker groups (two female, one male) and five never-smoker groups (three male, two female) were conducted (Table 1).

**Table 1:** Gender, smoking status and number of participants in each group

Group	Gender	Smoking Status	Number of participants
1	Female	Ever-smoker	5
2	Female	Ever-smoker	6
3	Male	Ever-smoker	5
4	Male	Never-smoker	5
5	Male	Never-smoker	5
6	Male	Never-smoker	4
7	Female	Never-smoker	5
8	Female	Never-smoker	6

### Materials

**Fig. 1:**
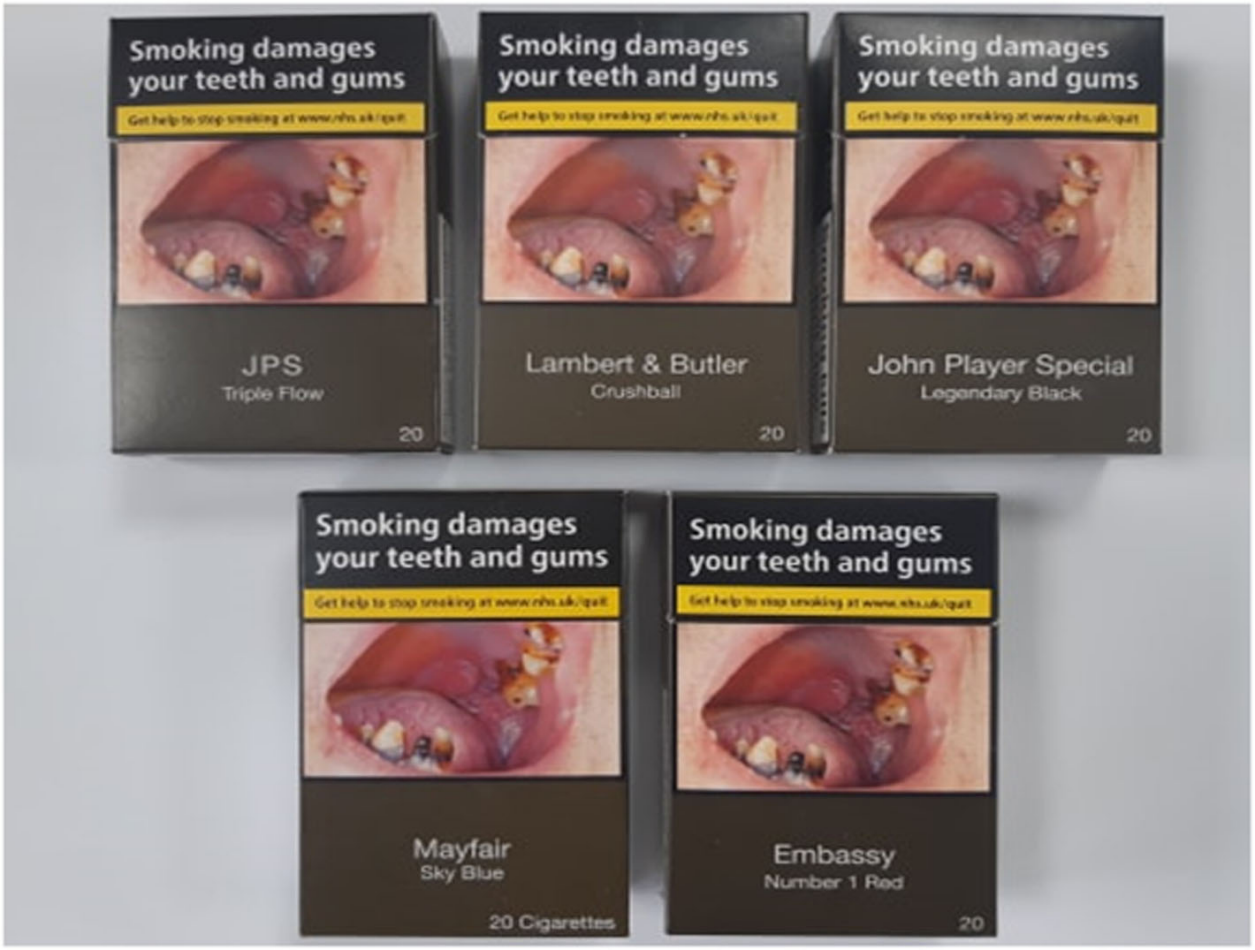
Straight-edged standardised packs with different brand variant names

Participants were exposed to, and given the opportunity to interact with, ten different standardised cigarette packs during the groups. The first five packs were straight-edged cigarette packs with flip-top lids, differing only by brand variant name (Fig. 1). The brand variants were selected to represent a range of cigarettes available in the UK market, which often include a colour within the name, mention filter innovation, or contain flavour changing capsules [26]. The following variants were included: *JPS Legendary Black, Mayfair Sky Blue, Embassy Number 1 Red, JPS Triple Flow* and *Lambert* and *Butler Crushball.*

**Fig. 2:**
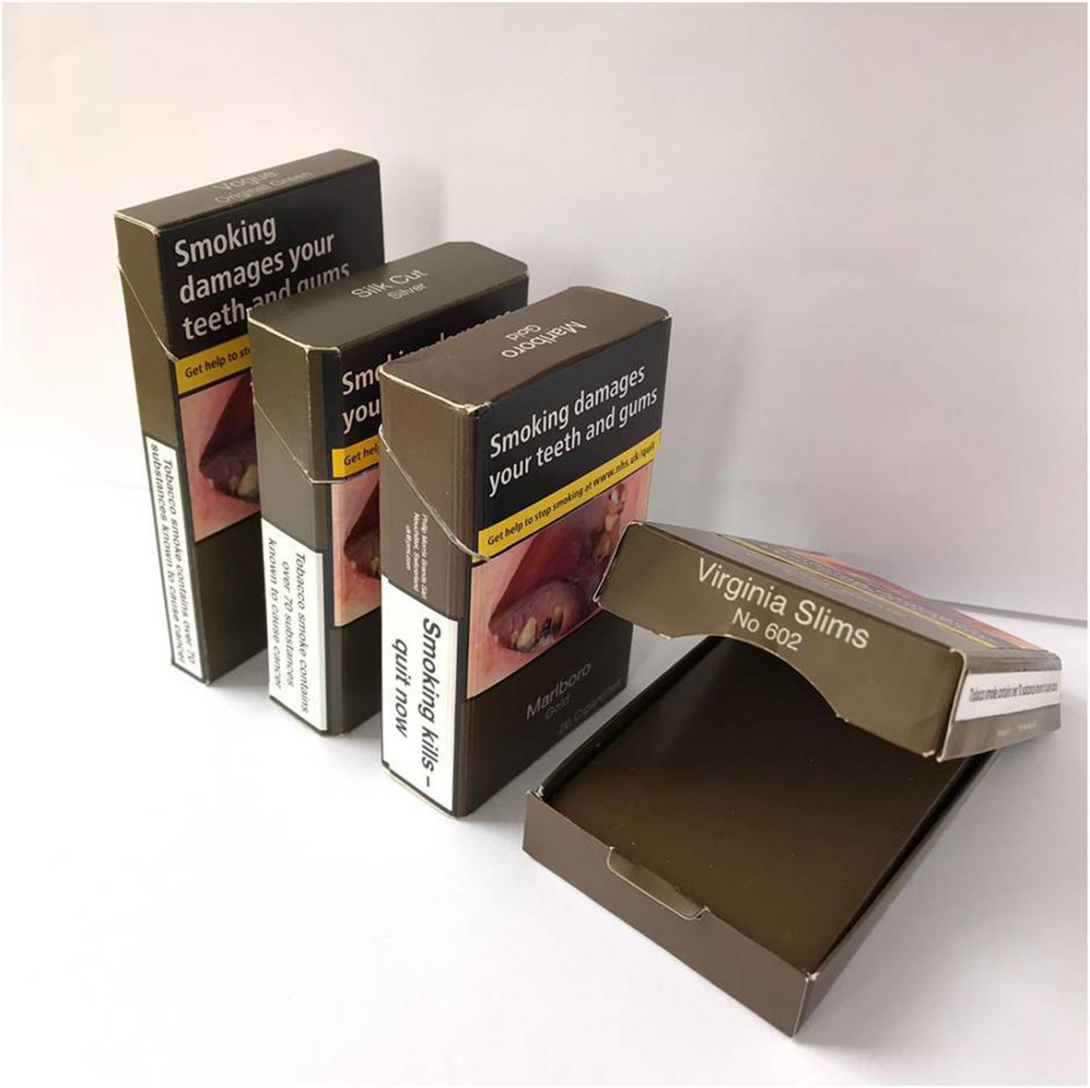
Standardised packs with different structures: From left to right rounded-edged pack, bevelled-edged pack, slim pack, shoulder box

The second set of five standardised cigarette packs, which varied in structure, consisted of a straight-edged pack (*Sterling Dual*), bevelled-edge pack (*Silk Cut Silver*), rounded-edge pack (*Marlboro Gold*), shoulder box (*Virginia Slims*), and a slim pack (*Vogue Green*) (Fig. 2). While shoulder box packs are permitted in the UK [10], no shoulder boxes had been identified on the UK market by the start of data collection. Consequently, the shoulder box used in this study was sourced from France, where standardised packs use the same drab brown colour. Although not currently sold, it was important to explore this feature since it is permitted under the UK legislation, and therefore packs using this opening method may still become available in the UK in the future.

All packs, including the shoulder box from France, carried the same pictorial and combined written warning to allow us to explore differences by brand variant name, or pack structure, without differences in warning design potentially confounding responses. The warning message, ‘Smoking damages your teeth and gums’, was considered most relatable to young people, given that it pertains to loss of attractiveness [27, 28].

### Procedure

Ethical approval was obtained from the University of Stirling’s General University Ethics Panel (GUEP273). Local councils were contacted to request permission to contact schools in their jurisdiction. Schools were approached by letter or email, followed up by a phone call. In the three schools that agreed to participate, prospective participants were informed about the study aims by the researcher or a teacher nominated by the school (e.g. those responsible for health and wellbeing education), and provided with an information sheet, privacy notice, consent forms, and pre-group questionnaires. All focus groups were conducted in classrooms at the school. At the start of each group, participants were reminded that their involvement was voluntary, they were free to withdraw at any time, their answers would be confidential, and all data provided would be anonymised. All groups lasted 30–45 min, with the duration of each group dictated by the length of the scheduled class period at each school. All groups were moderated by DM.

The focus groups began by exploring unprompted awareness of standardised packaging. Participants were asked where they see cigarette packs, who they see them with, what cigarette packs look like, and how they feel about them. Participants were then shown the first set of five packs and allowed to interact with them (Fig. 1). They were asked how they felt about them, what they thought about the colour, whether the packs made them feel differently about smoking, if they thought they were off-putting, how they would feel about using the packs and displaying them in public, how they thought people would react to them using the packs, whether they liked or disliked any of the packs, and their opinions of the warnings. Participants were then asked what they thought about the brand variant names and if they communicated anything about the cigarettes inside the pack.

The first five packs were removed prior to the next five packs, which varied in pack structure, being shown (Fig. 2). Participants were given time to look at the packs and encouraged to handle them, open them, and pass them around other group members. Time was given for comments to be made about the different pack features, with the moderator following up on these. Participants were then asked what they thought about the second set of five standardised packs. They were encouraged to look at the slim pack, packs with bevelled or rounded edges, and the shoulder box, alongside the standard straight-edged flip-top pack, thus providing an opportunity to comment on comparisons. They were also asked about their response to the warnings on these packs, to explore whether this was impacted by pack shape, opening style, and size.

At the end of each group, participants were debriefed about the study and provided with an information leaflet on the harms of smoking and sources of further advice. Participants were offered the opportunity to enter into a ballot to win a computer tablet for taking part.

### Analysis

All groups were audio recorded and transcribed verbatim by DM. The completed transcripts were then read several times by DM to facilitate familiarisation with the discussions. A thematic approach was used, which allowed for shared meaning or common attitudes across groups to be identified [29]. Using the key areas explored in the focus groups topic guide, a thematic coding framework was created in NVivo 11 by DM, following discussion with NC and CM who reviewed and commented on the transcribed group discussions, multiple times to refine the themes. The framework was based on the main topic areas covered in the topic guide given that the dominant themes were clearly linked to these. These were: Awareness of standardised packaging, Responses to standardised packs, Perceptions of brand variant name, Perceptions of pack structure, Health warning salience, and Perceived impact on smoking behaviour. Matrix coding in NVivo categorised themes by smoking status and gender to explore between group differences.

## Results

### Awareness of standardised packaging

When asked what cigarette packs look like, and before being shown any pack stimuli, most participants were generally aware of standardised packaging, with several mentioning that they are all the same colour and/or bland, e.g. “*It’s just like bland colours*” (Male ever-smoker)*.* There was awareness of standardised packaging in all three ever-smoker groups, and in two of the five never-smoker groups (one female, one male). Participants said that they frequently saw cigarettes packs, mainly as litter or used by other people (e.g. family or friends). Participants often mentioned the health warnings and recalled seeing certain images, including how smoking damages the lungs, heart, and other people, including young children and babies.

“They all look the same now do they not? They all had individual packaging and now it’s all the same” (Female ever-smoker).

“Compared to what they used to be they’re basic now, before they used to be pretty” (Female never-smoker).

“Well they have usually got a wee picture of an illness or something on it” (Male never-smoker).

### Responses to standardised packaging

When shown the first set of packs (Fig. 1), the consensus was that they all looked the same, being unappealing, disgusting, and off-putting. Some participants mentioned the lack of branding on the packs and that in general the packs would not really be noticed. The colour was often described as dirty, dull, or boring, with some commenting that they thought it reflected the harm caused by smoking. Several participants, mostly female, suggested that they would feel embarrassed about having one of the standardised packs or that they would not use them. The packs were viewed negatively in all groups.

“Nothing on its appealing, it’s just trying to stop you from smoking” (Female never-smoker).

“The colour of your lungs when you get lung cancer” (Female ever-smoker).

“They’re quite dark … so it’s not as if you’ll notice them much” (Male never-smoker).

“I would not open that in front of somebody … and imagine a wean [child] saw that or at a family gathering people saw that, quite embarrassing to have that” (Female never-smoker).

### Perceptions of brand variant name

Some participants considered the brand and variant names on the standardised packs they were shown to be appealing, particularly *Embassy Number 1 Red*, *Lambert and Butler Crushball*, *JPS Legendary Black* and *Mayfair Sky Blue*. The other brand name, *Sterling Dual*, was seldom mentioned. The favoured brand names were considered ‘*classy*’ and ‘*cool*’, particularly by males. Females were less likely to notice brand name, e.g. *“out of everything the brand name is the least stand out thing within the packet”* (Female never-smoker), or think that there were any differences, e.g. *“Unless you knew the names … like looking at them, you wouldn’t know which ones are better”* (Female ever-smoker). Several participants noted that the brand and variant name was the only thing left on the pack to create appeal, while others commented that the variant names with colours might be an attempt to remind people of the colours that were previously used on fully-branded packs.

“Lambert and Butler sounds quite classy” (Male ever-smoker).

“Well, Legendary Black, for example, is a bit like the Embassy one, it makes it sound cool and would maybe encourage you to buy it” (Male never-smoker).

“They are trying to encourage with the only one thing they can, like the brand more so than the cigarettes, they obviously now can’t have their fancy nice looking packet so they try with the name” (Female never-smoker).

“I think they are just reminiscing on their old colour, think they’re missing it [Mayfair Sky Blue]” (Male never-smoker).

### Perceptions of pack structure

When shown the second set of standardised packs (Fig. 2), participants often described the shoulder box as weird, cool, or different. It was generally viewed positively across groups. It was also thought it may be more expensive due to the opening style, which some participants suggested might encourage people to buy it.

“That is a stupid packaging to use because folk are gonna [going to] buy them cause [because] they look smart” (Female ever-smoker).

“I think that one [shoulder box] would be more expensive because it opens up differently” (Female ever-smoker).

“It’s like flip open … and then you think oh I can look cool” (Male never-smoker).

The slim pack was also viewed positively, with several, mostly female participants considering it the most attractive pack. It was also suggested that the cigarettes inside the slim pack might be less harmful, due to the thinness of the pack in comparison to the other packs. Some participants were curious about the cigarettes inside the pack and opened the pack. Among those that did, ever-smokers and males were more likely to suggest that they look nicer, compared them to confectionary cigarettes (i.e. sweets in the shape of a cigarette), or suggested that they would make them feel better about smoking, e.g. “*I wouldn’t feel as bad smoking that*” (Male never-smoker). Several male ever-smokers and never-smokers also noted that the slim pack would be easier to hide.

“I’d say that’s slightly more attractive than the thicker ones [packs], but then it wouldn’t attract me but it maybe is a bit prettier and thinner than the big massive one [pack]” (Female never-smoker).

“They look healthier cause [because] they are in a slim pack” (Male never-smoker).

“You feel like the bigger ones are more off-putting, cause like they’d be hard to hide in a pocket and stuff, but the slimmer ones you can just slide them in your pocket, it’s the same with a phone” (Male never-smoker).

Few participants noticed that two packs had bevelled or rounded edges. Most of the discussion around differences in edge type only arose after participants were prompted to compare these packs with the straight-edged pack. After being prompted, some participants suggested that these packs were more attractive or felt better to hold than a straight-edged pack, in particular males and female ever-smokers. Some female ever-smokers suggested that the Marlboro pack with the rounded edges was “*chic*” and a “*fashion statement*”, because they were packs that they had not seen before.

“They feel better in your hand” (Male never-smoker).

“Yeah, like, they are even like rounded as well instead of like straight or squared” (Female ever-smoker).

Several male never-smokers noted that because the second set of packs differed in structure there was more of a choice than if they were all identical, and that these structural features provided a way to create appeal.

“I still wouldn’t do it, but the diverse range of packets make it seem more appealing. Like the thinner one make it seem like its lighter and not as bad” (Male never-smoker).

“Makes you feel like you’ve got more of a choice, just based on the packaging” (Male never-smoker).

“I think they are trying to make it appeal in a different way than colour and names” (Male never-smoker).

### Health warning salience

Prior to seeing any pack stimuli, the health warnings were consistently one of the first things that participants recalled about cigarette packs. When shown the first set of five packs, the health warnings were considered clear, noticeable and believable, although within one female ever-smoker group it was felt that the warnings exaggerated the associated harms. Most participants, irrespective of smoking status, felt that the warnings reduced the appeal of smoking and agreed that they would put them off smoking. While participants most frequently commented on the pictorial warnings on the front and back of packs, there was mention of the text warnings on the secondary surfaces of the slim pack being smaller.

“Yeah, because they [health warnings] are most of the box [cigarette pack] it’s like a tiny name and then everywhere else it is big huge warnings so it’s like you can’t really avoid it” (Male never-smoker).

“Maybe if they saw tobacco smoke contains over 70 substances known to cause cancer, then probably would see that and think I better quit before it’s too late” (Male ever-smoker).

“I feel like the message on the side [slim pack] isn’t as clear because it is a lot thinner and smaller” (Female never-smoker).

### Perceived impact on smoking behaviour

Participants, in particular never-smokers, suggested that the standardised packs were off-putting, primarily because of the warnings, although the slimmer pack and shoulder box, and to a lesser extent the bevelled-edged and rounded-edged packs, were viewed as less of a deterrent. Several males suggested that they would feel uncomfortable about using these packs, with some stating that they would hide or conceal them.

“I would be quite awkward like I don’t know, like I wouldn’t be comfortable” (Male ever-smoker).

“Try and keep it hidden you would not want anyone to see your using them” (Male never-smoker).

“It’s quite off-putting innit [isn’t it], I don’t know why after seeing that person’s teeth [warning] you’d want to smoke” (Female never-smoker).

Concerning the impact on others, participants consistently stated that they thought the standardised packs, particularly the straight-edged packs, would have the least impact on established smokers, with addiction frequently offered as a reason. Nevertheless, several participants suggested that the packs would be off-putting for people thinking about taking up smoking, in particular young people.

“Doesn’t really stop their addiction, it’s just a packet, it would probably make them feel worse about smoking, but not stop them from smoking” (Male never-smoker).

“If they’ve just started and they seen that all the time it might off put them, like every single time they go to get a fag [cigarette] and they see that picture they might be like I don’t wanna [want to] smoke” (Female never-smoker).

## Discussion

We found that adolescent ever-smokers and never-smokers in Scotland perceived standardised cigarette packs negatively, with these packs considered to reduce the appeal of smoking. They often commented on how salient the health warnings on standardised packs were, considering them to be off-putting and suggesting that they may deter young people from starting smoking. These findings are consistent with previous research with adolescents [30–39].

Unlike in Australia and New Zealand, where cigarettes must be sold in straight-edged flip-top packs, standardised packaging legislation in the UK (and the other three European countries to have fully implemented standardised packaging) is less prescriptive. We found that a shoulder box, a slimmer pack, and packs with non-traditional shapes (i.e. with bevelled or rounded edges rather than straight edges) held greater appeal. For example, the shoulder box was viewed positively, being considered cool and different, and there was a preference for the slim pack among females. This is consistent with previous studies where young people are more likely to be drawn to packs with unique structures (e.g. bevelled-edged or slim packs) [13, 33, 40]. Although past research suggests that males view fully-branded slim packs negatively, primarily because of the slimness and feminine colour schemes [40, 41], we found that for some males standardised slim packs were viewed favourably as they were considered easier to conceal and appeared less harmful.

While any mention of taste, smell, flavour, or anything which may promote a product by creating an erroneous impression about its characteristics, is banned on standardised packs in the UK, tobacco companies recognise the increased importance of the brand variant name when all other branding is removed. In the UK, Australia, France and New Zealand, tobacco companies have continued to use brand and particularly variant name as a promotional tool post-standardised packaging [10, 26, 43, 44], changing existing variant names (e.g. ‘Silver’ to ‘Silver Stream’) or introducing new variants (e.g. ‘Silver Fine Scent’, ‘Master Blend Blue’, ‘Black Alaska’). Consistent with past research [37, 41, 42], we found that among males in particular brand variant names on otherwise identical packs (e.g. JPS Legendary Black) can still have an appeal function. In addition, it was suggested that colour descriptors, which are frequently used on standardised packs [26, 44], may be intended to invoke memories of the colour that was previously used for fully-branded packaging. As past research has found that variant names using colour descriptors can shape product perceptions [37 , 41, 42], governments planning to introduce standardised packaging may prefer to ban colour descriptors on packs.

Concerning limitations, only a small number of ever-smokers were recruited. This may, in part, be due to the voluntary basis of the study, and as the study took place in a school setting adolescents may have felt uncomfortable indicating that they had tried smoking. This limits our understanding of the response of young smokers to standardised packaging. While teachers were not present in the groups, being part of a peer group may have also lead some participants to provide socially desirable responses. While it was our intention to focus on adolescents aged 16 and 17, and qualitative research is not intended to be generalisable, we nevertheless only provide an insight into a very narrow age group. As there remains a lack of consumer research post-standardised packaging in the UK [45], there are a number of avenues for future research. Exploring perceptions of standardised packs using quantitative methods with young people and what association (if any) pack structure has with smoking susceptibility would be of value. Future research could also explore the effect, if any, of removing colour descriptors from the variant name, or removing the entire brand variant name from packs and replacing it with a number, as proposed in Turkey [13].

## Conclusions

While our study found that the warnings on standardised packs were salient and that these packs were generally off-putting, the legislation in the UK allows tobacco companies to continue to use pack structure (shape, size and opening style) and brand variant name to differentiate brands and create appeal. Additional research exploring how this may affect the intended goals of standardised packaging is warranted, and would be informative for countries planning to introduce this policy.

## Data Availability

The datasets used and/or analysed during the current study are available from the corresponding author on reasonable request.
